# Impact of COVID-19 pandemic on prostate cancer outcomes at an uro-oncology referral center

**DOI:** 10.1590/S1677-5538.IBJU.2022.0393

**Published:** 2023-02-05

**Authors:** Guilherme Miranda Andrade, Lucas Sesconetto, Rafael Benjamim Rosa da Silva, Gabriela Guimarães Rodrigues dos Santos, Paulo Priante Kayano, Willy Baccaglini, Murilo Borges Bezerra, Bianca Bianco, Gustavo Caserta Lemos, Arie Carneiro

**Affiliations:** 1 Departamento de Urologia Hospital Israelita Albert Einstein São Paulo SP Brasil Departamento de Urologia, Hospital Israelita Albert Einstein - HIAE, São Paulo, SP, Brasil;; 2 Faculdade Israelita de Ciências da Saúde Albert Einstein São Paulo SP Brasil Faculdade Israelita de Ciências da Saúde Albert Einstein, São Paulo, SP, Brasil

**Keywords:** Prostatic Neoplasms, COVID-19, Prostatectomy

## Abstract

**Introduction:**

To evaluate the possible effects of the coronavirus disease 2019 (COVID-19) pandemic on the oncologic results of patients with prostate cancer regarding clinical staging, presence of adverse pathological outcomes, and perioperative complications.

**Materials and methods:**

This retrospective study included patients who underwent radical prostatectomy. The time between biopsy and surgery, staging tests, final histopathological evaluation after surgery, lymphadenectomy rate, postoperative complications, and prostatic specific antigen (PSA) levels (initial and 30 days after surgery) were analyzed and compared in a group of patients before and during the pandemic period.

**Results:**

We included 226 patients: 88 in the pre-pandemic period and 138 during the pandemic period. There was no statistically significant difference in mean age, body mass index, ASA, pathological locally advanced disease, the proportion of patients who underwent lymphadenectomy, and ISUP grade in the biopsy between the groups. Positive surgical margins, prostatic extracapsular extension, and PSA levels at 30 days were also similar between the groups. The mean time between medical consultation and surgery was longer in the pandemic period than in the pre-pandemic (124 vs. 107 days, p<0.001), and the mean time between biopsy and medical consultation (69.5 days vs. 114 days, p<0.001) and between biopsy and surgery (198.5 days vs. 228 days, p=0.013) was shorter during the pandemic. The incidence of severe early and late perioperative complications was similar between the periods.

**Conclusions:**

There was no delay between diagnosis and treatment at our institution during the COVID-19 pandemic period. No worsening of the prostate cancer features was observed.

## INTRODUCTION

The first patient with coronavirus disease 2019 (COVID-19) in São Paulo, Brazil was confirmed on February 26, 2020. The number of confirmed cases grew in a classical exponential curve, with a rapid rate per day (~25%) comparable to that observed in other countries ( 1 ). Within 23 days of the first case, emergency public health decisions were taken to protect the vulnerable, minimize its impact on healthcare, and reduce community transmission ( 2 , 3 ). On March 11, 2020, the World Health Organization declared COVID-19 a pandemic ( 1 ). The COVID-19 pandemic is the most recent and largest pandemic we have experienced in recent decades. Thus, the health system is undergoing profound changes related to the use of resources and distribution of health inputs ( 4 - 6 ).

Electing a patient for a urological surgical procedure within the context of the pandemic involves great responsibility because it increases the risk of contagion for the patient, healthcare professionals, and other patients ( 7 , 8 ). In view of the high demand for hospital beds and the relocation of health professionals to face the disease worldwide, elective surgeries have been postponed or canceled in favor of the operation of high-risk patients, urgencies, or emergencies ( 9 - 13 ). From a urological surgery perspective, many questions have arisen regarding the immediate and long-term care of patients.

There is some evidence in the literature that suggests that delays in the treatment of patients with prostate cancer (PCa) lead to higher rates of adverse factors in the final pathology (Gleason score, surgical margin, and extracapsular prostate extension) ( 14 ). Thus, identifying the impact of delayed diagnosis of PCa during the COVID-19 pandemic is essential for the organization of uro-oncology services and for dealing with future pandemics ( 15 ).

Based on these findings, we aimed to evaluate the possible detrimental effects of the COVID-19 pandemic on the oncologic outcomes of patients with PCa regarding clinical staging, presence of adverse pathologic outcomes, and perioperative complications compared with the pre-pandemic period.

## MATERIAL AND METHODS

The design, analysis, interpretation of data, drafting, and revisions followed the Helsinki Declaration and the strengthening of the reporting of observational studies in epidemiology (STROBE) statement, which is available through the enhancement of the quality and transparency of health research (EQUATOR) network (www.equator-network.org). The study design was approved by the local independent Research Ethics Committee (approval code: CAAE 54077521.4.0000.0071). The requirement for informed consent was waived by the research ethics committee.

This retrospective, observational study included patients who underwent treatment for non-metastatic PCa with curative intent from June 2019 to June 2021.The study was conducted in a public hospital in Sao Paulo that is managed by the *Hospital Israelita Albert Einstein* as a result of a public-private partnership with the City Hall of São Paulo. This hospital is associated with the Medical Residency Program in Urology of the *Faculdade Israelita de Ciências da Saúde Albert Einstein* (Medical School). Patients were divided into two groups: pre-pandemic (November, 2018 to February, 2020) and pandemic (from March, 2020 to June, 2021).

### Patients

The inclusion criterion was patients who underwent treatment for non-metastatic PCa with curative intent through radical prostatectomy with or without lymphadenectomy. All patients were diagnosed with PCa after a change in screening, and subsequent biopsies were performed at a primary health service. After diagnosis, patients were referred to our specialized center for treatment. Patients with PCa undergoing treatment without a curative proposal or treatment other than radical prostatectomy, and patients with synchronous or metachronous neoplasms were excluded from the study.

### Data Collection

Data were collected from the electronic medical records of each patient, including age, initial prostatic specific antigen (PSA) levels, lymphadenectomy, International Society of Urological Pathology (ISUP) grade, surgical margin, prostatic extracapsular extension found in the surgical specimen obtained after radical prostatectomy, PSA level at 30 days, time between biopsy and first medical consultation (medical appointment in our tertiary center when the patient had already undergone biopsy and the diagnosis of PCa was established by the primary healthcare center), interval between first medical consultation and surgery, total time between biopsy and surgery, and severe complications in the early and late perioperative period of radical prostatectomy (Clavien Dindo III or IV).

## Statistical Analysis

Data analysis was used to determine differences between the groups of patients who attended before and during the COVID-19 pandemic. Categorical data were analyzed using absolute and relative frequencies. Numerical data were tested for normal distribution using Shapiro–Wilk test, and none of the variables presented a normal distribution. All data were presented as median and interquartile range (IQR). Missing numerical data were treated with median imputation if the missing values did not exceed 10% of the total observations. No policy was implemented for missing categorical data. Mann–Whitney U test was used for bivariate comparisons between numerical variables, and the Chi-squared test was used for categorical data and comparisons between numerical and categorical data. Bonferroni correction was used for groups with more than two categories when differences were observed. The significance level was set at P<0.05. The analyses were performed using Python™, version 3.8 on the Jupyter Notebook, version 6.4.8.

## RESULTS

**Figure 1:**
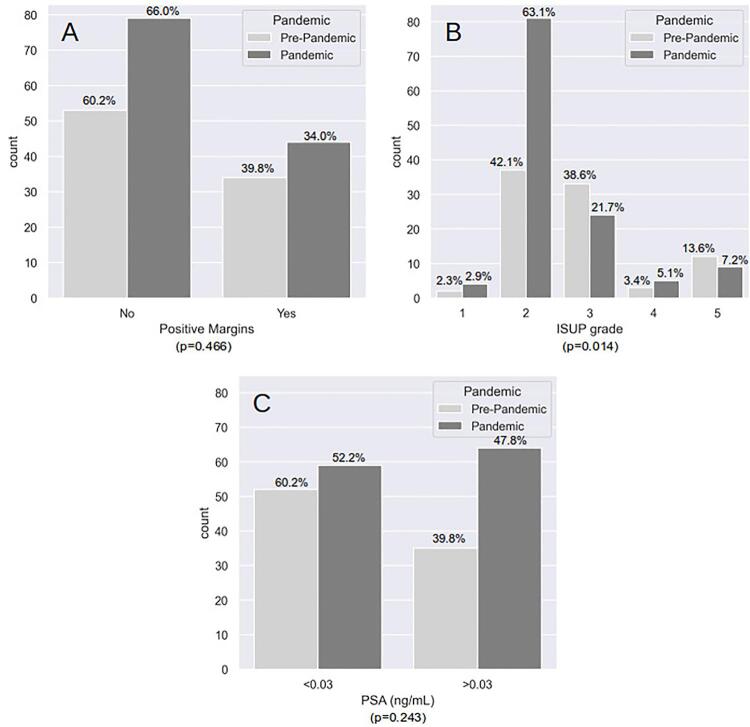
Pathological characteristics of patients studied. (A) Positive Margin, (B) ISUP grade according to final pathology, and (C) Positive PSA level in 30 days after surgery.

A total of 226 patients were included in this study: 88 in the pre-pandemic period and 138 in the pandemic period. The general characteristics of the patients and comparisons between the groups are shown in Table-1 and Figure-1 .

**Table 1:** Baseline demographic and pathological characteristics of patients studied.

Variables	Pre-Pandemic	Pandemic	p-value
Patients (n, %)	88 (38.9%)	138 (61.1%)	---
Age (years)	64.0 [58.0-69.0]	63.0 [59.0-67.0]	0.373 ^a^
BMI (Kg/m^2^)	27.3 [24.5-28.9]	27.3 [25.3-29.9]	0.249 ^a^
**ASA (n, %)**			
1	10 (11.4%)	10 (7.3%)	
2	64 (72.7%)	114 (82.6%)	0.208 ^b^
3	14 (15.9%)	14 (10.1%)	
Initial PSA (ng/dL)	10.1 [6.0-17.2]	7.7 [5.4-11.3]	0.007 ^a^
Neoadjuvant androgen deprivation (n, %)	1 (1.1%)	15 (10.9%)	0.011 ^b^
Adjuvant Radiotherapy (n, %)	33 (37.5%)	21 (15.2%)	<0.001 ^b^
**Prostatectomy (n, %)**			
Open	56 (63.6%)	107 (77.5%)	0.023 ^b^
Videolaparoscopic	32 (36.4%)	31 (22.5%)	
Lymphadenectomy (n, %)	40 (45.5%)	76 (55.1%)	0.203 ^b^
Pathological locally advanced disease (pT3–4) (n, %)	33 (37.5%)	34 (24.6%)	0.081 ^b^
**D’amico Risk Group**			
Low Risk	10 (11.4%)	16 (11.6%)	
Intermediate Risk	42 (47.7%)	75 (54.4%)	0.564 ^b^
High Risk	36 (40.9%)	47 (34.0%)	
**ISUP_Biopsy (n, %)**			
1	21 (23.8%)	26 (18.9%)	
2	40 (45.5%)	71 (51.4%)	
3	20 (22.7%)	27 (19.6%)	0.739 ^b^
4	4 (4.5%)	10 (7.2%)	
5	3 (3.5%)	4 (2.9%)	
**ISUP Surgery (n, %)**			
1	2 (2.3%)	4 (2.9%)	
2	37 (42.1%)	87 (63.1%)	
3	34 (38.6%)	30 (21.7%)	0.014 ^b, c^
4	3 (3.4%)	7 (5.1%)	
5	12 (13.6%)	10 (7.2%)	
**ISUP >3 Surgery (n, %)**	15 (17.0%)	17 (12.3%)	0.425 ^b^
Positive surgical margin (n, %)	35 (39.8%)	47 (34.0%)	0.466 ^b^
Prostatic extracapsular extension (n, %)	38 (43.2%)	43 (31.1%)	0.089 ^b^
Positive PSA level in 30 days	35 (39.8%)	66 (47.8%)	0.293 ^b^
Time between biopsy and medical consultation (days)	114.0 [90.0-176.3]	69.5 [42.5-118.5]	<0.001 a
Time between medical consultation and surgery (days)	107.0 [64.0-114.3]	124.0 [76.0-213.0]	<0.001 ^a^
Time between biopsy and surgery (days)	228.0 [185.5-323.75]	198.5 [132.5-291.0]	0.013 ^a^
Time of anesthesia (minutes)	250.0 [241.5-250.0]	255.0 [210.0-300.0]	0.043 ^a^
Time of surgery (minutes)	200.0 [199.0-200.0]	202.5 [165.0-240.0]	0.084 ^a^
Severe early perioperative complications (n, %)	5 (5.7%)	8 (5.8%)	0.797 ^b^
Severe late perioperative complications (n, %)	2 (2.3%)	5 (3.6%)	0.858 ^b^

* Qualitative variables were presented by absolute and relative frequency, and quantitative variables by median and interquartile range.

ASA = American Society of Anesthesiology score; BMI = Body mass index; ISUP = International Society of Urological Pathology; PSA = Prostate Specific Antigen.

a = Mann-Whittney U test; b = Chi-Square test; c =Post hoc analysis showed statistical significant difference regarding the proportion of ISUP grade 2 (p<0.001) between pre and pandemic groups.

The variables were compared using the chi-square test.

There were no differences in age, body mass index (BMI), American Society of Anesthesiologists Classification score (ASA), pathological locally advanced disease, proportion of patients who underwent lymphadenectomy, and ISUP grade found in the transrectal biopsy between the groups. The initial PSA levels were significantly higher in the pre-pandemic group (10.1 ng/dL vs. 7.7 ng/dL, p=0.007). Most patients in both groups presented intermediate D’amico risk, and the proportion of the high-risk group was similar pre- and during the pandemic (40.9% vs. 34%, p=0.564). The number of patients who underwent neoadjuvant androgen deprivation was higher in the pandemic group (10.9% vs. 1.1%, p=0.011), whereas the number of patients who underwent adjuvant radiotherapy was significantly higher in the pre-pandemic group (37.5% vs. 15.2%, p≤0.001).

The type of prostatectomy differed between the pre- and pandemic groups (open, 63.6% vs. 77.5%; and video laparoscopic, 36.4% vs. 22.5%; p=0.023). During the pre-pandemic period, major complications (Clavien Dindo 3 and 4) occurred in five patients (5.7%): compartment syndrome requiring fasciotomy, two urinary leaks (one with a cystoscopy procedure for diagnostic confirmation), one patient was referred to the intensive care unit because of altered mental status and confusion after surgery, and a ureteral lesion during lymphadenectomy was visualized and sutured during the surgery. During the pandemic period, major complications were present in eight patients (5.8%): two rectal lesions, five patients with bleeding referred to the intensive care unit to control blood pressure, and one patient lost the bladder catheter and needed a new catheterization.

The histological characteristics according to the final pathology showed differences in the ISUP grade between the groups. Although there was no difference in ISUP >3, post hoc analysis showed a statistically significant difference in the proportion of ISUP grade 2 (42.1% vs. 63.1%, p<0.001) between the pre- and pandemic groups. Positive surgical margins, prostatic extracapsular extension, and positive PSA levels at 30 days were similar between the groups. Although the mean time between medical consultation and surgery was longer during the pandemic period than during the pre-pandemic period (124 days vs. 107 days, p<0.001), the mean time between biopsy and medical consultation (69.5 days vs. 114 days, p<0.001) and biopsy and surgery (198.5 days vs. 228 days, p=0.013) was significantly shorter during the pandemic period. Albeit the mean time of anesthesia was significantly higher in the pandemic group (250.0 min vs. 255.0 min, p=0.043), the mean time of surgery, and severe early and late perioperative complications were similar during the pre- and pandemic periods.

## DISCUSSION

To the best of our knowledge, this is the first study to evaluate the possible detrimental effects of the COVID-19 pandemic on oncological treatment outcomes in patients with PCa in South America.

According to the Brazilian Society of Urology, the number of prostate biopsies performed in Brazil decreased from 2019 to 2020, and there was a delay in performing biopsies and diagnosing prostatic diseases. In the state of São Paulo, this decrease was 6%, but in other states, it reached 90% ( 16 ). These numbers are probably due to better screening and treatment of patients with PCa in São Paulo than in other regions of Brazil. In the present study, we observed lower initial PSA levels during the pandemic period (7.7 ng/dL vs. 10.1 ng/dL) and also a shorter time between the biopsy (diagnosis) and first consultation (114 days vs. 69.5 days), possibly as a reflection of PCa screening that has been improving over the years, in spite of the COVID-19 outbreak.

The teaching hospital of Albert Einstein Medical School, associated with the medical residence in Urology, underwent restructuring due to the overoccupancy of hospital vacancies during the pandemic. There was a reduction and some suspension of elective procedures, such as prostatectomies and biopsies, which caused delays in the treatment of patients with cancer. Thus, there was a change in the treatment strategy (patients who would undergo surgery were referred for radiotherapy), and some patients underwent initial hormone block therapy to receive definitive treatment (surgery or radiotherapy). The same redistribution of patients was described by Korkes et al. ( 17 ), who observed that an increase in adjunctive advanced disease occurred during the years of COVID-19. This might indicate that patients were preferably sent for neoadjuvant advanced disease following the recommendations of the guidelines during the COVID-19 pandemic.

During the pandemic, there was an increase in the referral of patients with PCa to our institution. Consequently, the number of patients undergoing radical prostatectomy was 56% higher during this period (88 during the pre-pandemic period and 138 during the pandemic period). This movement of greater referral of patients with cancer to our center is the result of a complex infrastructure and specialized multidisciplinary staff, which involves oncologists, urologists, radiologists, radiotherapists, and advanced technology to treat these patients. Despite this absolute increase in the number of patients, the surgeons who performed the surgery, the surgical technique used, the material used in the surgeries, and the postoperative care were identical in both groups, which would not justify the difference in the results between them.

In the present study, the number of patients who underwent neoadjuvant androgen deprivation was higher in the pandemic group (10.9% vs. 1.1%), whereas the number of patients who underwent adjuvant radiotherapy was significantly higher in the pre-pandemic group (37.5% vs. 15.2%). The higher rate of salvage radiotherapy in the pre-pandemic period can be explained by the longer time that these groups experienced between surgery and follow-up in comparison with the shorter time in the pandemic group to relapse of prostate cancer. Therefore, the patients operated during the pandemic may still be under the risk of presenting biochemical recurrence during the following years. In turn, the difference in the proportion of neoadjuvant androgen deprivation after and during the pandemic can also be explained by the strategy of forwarding patients to this treatment to postpone definitive treatment during the period when elective surgeries were canceled, and the radiotherapy service was already overcrowded. This highlights the importance of the organization of health services in the management of pandemics.

Despite the pandemic, we observed that the time between biopsy and surgery was significantly shorter during the pandemic period (198.5 days vs. 228 days). The time between biopsy and surgery has been extensively discussed in the literature to better understand if and how the delay could affect the oncological results. Berger et al. ( 14 ) reported that delays of 150 days in the low-risk group and 30 days in the high-risk group lead to worse pathological outcomes. Similarly, Auffenberg et al. ( 4 ) observed in a prospective cohort study that patients who underwent delayed prostatectomy were more likely to have a Gleason score of 7 or greater than those who underwent immediate surgery (69.2% vs. 48.8%).

On the other hand, the impact of delayed prostatectomy on pathological outcomes is questionable by some studies, even in high-risk patients ( 18 - 23 ). A large cohort study found that among 32,184 patients, delay up to 6 months performing radical prostatectomy did not lead to an increase in the incidence of positive surgical margins, positive lymph nodes, or increases in T3 and T4 cases ( 24 ). Likewise, a retrospective study of 128,062 men with intermediate- and high-risk PCa treated with radical prostatectomy in the American National Database did not show a significant difference in the odds of adverse pathology, upgrading, node-positive disease, or post-radical prostatectomy secondary treatments between men treated with immediate radical prostatectomy and any level of delay up to 12 months ( 25 ).

Several recommendations have recently been published to guide the management of urological conditions during these troubled times ( 26 , 27 ). Based on the findings of the aforementioned studies, accumulating evidence supports the idea that radical prostatectomy can be safely postponed when the availability of healthcare resources is limited ( 20 , 28 , 29 ).

The proportion of video-laparoscopic prostatectomies performed during the COVID-19 period was lower than that during the pre-pandemic period (36.4% vs. 22.5%). The operating room environment has historically been prepared to prevent infection by agents transmitted mainly through contact with blood and body fluids. However, aerosol protection was not part of this routine. Surgical centers are structured in a closed area with little air exchange and generally no negative pressure. These conditions favor the transmission of SARS-CoV-2 among patients, members of the surgery team, and employees of the sector. Video-laparoscopic surgery is based on the creation of an intracavitary, peritoneal, or extraperitoneal space with carbon dioxide insufflation, which raises concerns about the possibility of SARS-CoV-2 transmission via this route ( 30 ). Thus, especially in the first months of the pandemic, the concern of contamination during laparoscopic surgeries may have impacted the increase in open prostatectomy. Nevertheless, the incidence of major complications in the pre- and pandemic periods was similar (5.7% vs. 5.8%) and unrelated to the COVID-19 pandemic.

The rate of positive PSA results (greater than 0.03 ng/dL) before and during the COVID-19 pandemic was not statistically different (39.8% vs. 47.8%). In the present study, the first PSA level was often assessed in a period of less than 30 days, so the positive value in many patients is in fact a PSA level in the decline of the half-life curve.

In agreement with Oderda et al. ( 29 ), we also believe that the centralization of uro-oncological activity in referral centers is essential to guarantee safe and high-quality treatments, and even more so in times of crisis, such as the COVID-19 pandemic. No delay between diagnosis and surgery was observed in our study compared to the procedures of the pre-pandemic period; no significant difference in terms of main pathologic features was observed, likely as a consequence of our role as a referral center.

Concerning study limitations, our study was performed at a single center, and the short time span of the study might have hampered the evaluation of the effects of delayed screening due to COVID-19. In addition, the physical structure and clinical staff of our hospital have grown gradually over the last three years, so that even during the pandemic, there was a greater number of patients in our clinics and, consequently, resulted in an increase in prostatectomies performed during the pandemic. All patients were screened for SARS-CoV-2 using rapid antigen tests 48 h prior to surgery during the COVID-19 pandemic. Surgery was postponed for at least six weeks for those who tested positive. No modification was required for the anesthesia protocol that remained a general anesthesia with endotracheal intubation and spinal block. Even though the number of rooms available decreased by 25-70% between 2020 and 2021, surgeries were still performed in the same operating room usually designated to the team. Nonetheless, oncological surgeries were prioritized compared to other cases. Due to the drastic shortage of SARS-CoV-2 rapid antigen tests, asymptomatic patients were not retested after surgery. Therefore, it was not possible to obtain data regarding post-treatment COVID-19 infection rates in this sample. Finally, protective procedures were adopted by all professionals according to the current protocols.

## CONCLUSIONS

It is noteworthy that there was an absence of delay in the treatment of PCa at our institution during the COVID-19 pandemic, as well as no worsening of pathological features. This study reinforces that even with the challenges and limitations imposed by the pandemic outbreak, well-structured facilities allied to an agile management are of paramount importance for a healthcare center to provide in-time treatment for prostate cancer preserving adequate clinical and perioperative outcomes.

## References

[1] World Health Organization (2020). Coronavirus disease 2019 (COVID-19).

[2] Garcia PJ, Alarcón A, Bayer A, Buss P, Guerra G, Ribeiro H (2020). COVID-19 Response in Latin America. Am J Trop Med Hyg.

[3] Serdan TDA, Masi LN, Gorjao R, Pithon-Curi TC, Curi R, Hirabara SM (2020). COVID-19 in Brazil: Historical cases, disease milestones, and estimated outbreak peak. Travel Med Infect Dis.

[4] Auffenberg GB, Linsell S, Dhir A, Myers SN, Rosenberg B, Miller DC (2016). Comparison of Pathological Outcomes for Men with Low Risk Prostate Cancer from Diverse Practice Settings: Similar Results from Immediate Prostatectomy or Initial Surveillance with Delayed Prostatectomy. J Urol.

[5] Yang Y, Peng F, Wang R, Yange M, Guan K, Jiang T (2020). The deadly coronaviruses: The 2003 SARS pandemic and the 2020 novel coronavirus epidemic in China. J Autoimmun.

[6] Zumla A, Niederman MS (2020). Editorial: The explosive epidemic outbreak of novel coronavirus disease 2019 (COVID-19) and the persistent threat of respiratory tract infectious diseases to global health security. Curr Opin Pulm Med.

[7] Puliatti S, Eissa A, Eissa R, Amato M, Mazzone E, Dell’Oglio P (2020). COVID-19 and urology: a comprehensive review of the literature. BJU Int.

[8] Steward JE, Kitley WR, Schmidt CM, Sundaram CP (2020). Urologic Surgery and COVID-19: How the Pandemic Is Changing the Way We Operate. J Endourol.

[9] Naspro R, Da Pozzo LF (2020). Urology in the time of corona. Nat Rev Urol.

[10] Tefik T, Guven S, Villa L, Gokce MI, Kallidonis P, Petkova K (2020). Urolithiasis Practice Patterns Following the COVID-19 Pandemic: Overview from the EULIS Collaborative Research Working Group. Eur Urol.

[11] Gravas S, Bolton D, Gomez R, Klotz L, Kulkarni S, Tanguay S (2020). A Global Perspective and Snapshot Analysis. J Clin Med.

[12] Gorgen ARH, Diaz JO, Silva AGT, Paludo A, Oliveira RT, Tavares PM (2021). The impact of COVID-19 pandemic in urology practice, assistance and residency training in a tertiary referral center in Brazil. Int Braz J Urol.

[13] Prezotti JA, Henriques JVT, Favorito LA, Canalini AF, Machado MG, Brandão TBV (2021). Impact of COVID-19 on education, health and lifestyle behaviour of Brazilian urology residents. Int Braz J Urol.

[14] Berg WT, Danzig MR, Pak JS, Korets R, RoyChoudhury A, Hruby G (2015). Delay from biopsy to radical prostatectomy influences the rate of adverse pathologic outcomes. Prostate.

[15] Teixeira TA, Bernardes FS, Oliveira YC, Hsieh MK, Esteves SC, Duarte-Neto AN (2021). SARS-CoV-2 and Multi-Organ damage - What men’s health specialists should know about the COVID-19 pathophysiology. Int Braz J Urol.

[16] Dados do Ministério da Saúde mostram queda no número de consultas, cirurgias e internações relacionados a doenças da próstata. Sociedade Brasileira de Urologia. Novembro Azul 2021.

[17] Korkes F, Smaidi K, Timoteo F, Glina S (2022). Recommendations for prostate cancer diagnosis and treatment during COVID-19 outbreak were not followed in Brazil. Int Braz J Urol.

[18] Diamand R, Ploussard G, Roumiguié M, Oderda M, Benamran D, Fiard G (2021). Timing and delay of radical prostatectomy do not lead to adverse oncologic outcomes: results from a large European cohort at the times of COVID-19 pandemic. World J Urol.

[19] Tosoian JJ, Sundi D, Trock BJ, Landis P, Epstein JI, Schaeffer EM (2016). Pathologic Outcomes in Favorable-risk Prostate Cancer: Comparative Analysis of Men Electing Active Surveillance and Immediate Surgery. Eur Urol.

[20] Patel P, Sun R, Shiff B, Trpkov K, Gotto GT (2019). The effect of time from biopsy to radical prostatectomy on adverse pathologic outcomes. Res Rep Urol.

[21] Ahmad AE, Richard PO, Leão R, Hajiha M, Martin LJ, Komisarenko M (2020). Does Time Spent on Active Surveillance Adversely Affect the Pathological and Oncologic Outcomes in Patients Undergoing Delayed Radical Prostatectomy?. J Urol.

[22] Morini MA, Muller RL, Castro PCB, Souza RJ, Faria EF (2018). Time between diagnosis and surgical treatment on pathological and clinical outcomes in prostate cancer: does it matter?. World J Urol.

[23] Laukhtina E, Sari Motlagh R, Mori K, Quhal F, Schuettfort VM, Mostafaei H (2021). Oncologic impact of delaying radical prostatectomy in men with intermediate- and high-risk prostate cancer: a systematic review. World J Urol.

[24] Xia L, Talwar R, Chelluri RR, Guzzo TJ, Lee DJ (2020). Surgical Delay and Pathological Outcomes for Clinically Localized High-Risk Prostate Cancer. JAMA Netw Open.

[25] Ginsburg KB, Curtis GL, Timar RE, George AK, Cher ML (2020). Delayed Radical Prostatectomy is Not Associated with Adverse Oncologic Outcomes: Implications for Men Experiencing Surgical Delay Due to the COVID-19 Pandemic. J Urol.

[26] Ribal MJ, Cornford P, Briganti A, Knoll T, Gravas S, Babjuk M (2020). European Association of Urology Guidelines Office Rapid Reaction Group: An Organisation-wide Collaborative Effort to Adapt the European Association of Urology Guidelines Recommendations to the Coronavirus Disease 2019 Era. Eur Urol.

[27] Heldwein FL, Loeb S, Wroclawski ML, Sridhar AN, Carneiro A, Lima FS (2020). A Systematic Review on Guidelines and Recommendations for Urology Standard of Care During the COVID-19 Pandemic. Eur Urol Focus.

[28] Campi R, Amparore D, Capitanio U, Checcucci E, Salonia A, Fiori C (2020). Assessing the Burden of Nondeferrable Major Uro-oncologic Surgery to Guide Prioritisation Strategies During the COVID-19 Pandemic: Insights from Three Italian High-volume Referral Centres. Eur Urol.

[29] Oderda M, Soria F, Rosi F, Calleris G, Mazzoli S, Giordano A (2022). COVID-19 pandemic impact on uro-oncological disease outcomes at an Italian tertiary referral center. World J Urol.

[30] Zheng MH, Boni L, Fingerhut A (2020). Minimally Invasive Surgery and the Novel Coronavirus Outbreak: Lessons Learned in China and Italy. Ann Surg.

